# Association between Visceral Fat and Bone Mineral Density in Both Male and Female Patients with Adult Growth Hormone Deficiency

**DOI:** 10.1155/2020/5079625

**Published:** 2020-07-05

**Authors:** Linman Li, Li Zhong, Xiaoya Zheng, Wenyi You, Yunting Wang, Jihui Yu, Xun Wu, Wei Ren, Gangyi Yang

**Affiliations:** ^1^Department of Health Management Center, The First Affiliated Hospital of Chongqing Medical University, Chongqing 400016, China; ^2^Department of Endocrinology, The First Affiliated Hospital of Chongqing Medical University, Chongqing 400016, China; ^3^Infectious Diseases Department, The People's Hospital of Yubei District of Chongqing City, Chongqing 401120, China; ^4^Department of Endocrinology, The Second Affiliated Hospital, Chongqing Medical University, Chongqing 400010, China

## Abstract

**Aim:**

Adult growth hormone deficiency (AGHD) is associated with an increased risk of fractures. The interactions between various body composition and bone are known to be complex in nature. However, very few studies have examined this crosstalk in AGHD. In this study, we sought to investigate the relationship between various parameters of body composition and bone mineral density (BMD) as well as determine the role of visceral fat in determining the bone mass in patients with AGHD.

**Methods:**

We conducted a cross-sectional study on 57 patients with AGHD. Anthropometry, biochemistry, and analysis of body composition and BMD were performed according to standard protocols. Male and female patients were classified into those with osteoporosis and those without osteoporosis (normal subjects and patients with osteopenia). Further, we analyzed the correlation between the BMD and measurements obtained for various body composition parameters in male and female AGHD patients.

**Results:**

Our findings indicated that among female AGHD patients, those with osteoporosis had a significantly higher levels of fat mass (FM) and visceral adipose tissue mass (VATM) (both, *P* < 0.05) than those without osteoporosis. Further, Pearson correlation analysis showed that the values of age, body mass index (BMI), FM, and VATM correlated negatively with BMD in women with AGHD (all *P* < 0.05); however, this association was not noted in men. After adjusting for the other covariates, VATM was found to be independently correlated with the BMD in female patients with AGHD.

**Conclusions:**

A close correlation was noted between VATM and BMD in female patients with AGHD.

## 1. Introduction

Osteoporosis is a metabolic bone disease that is characterized by decreased bone mass and a high risk of fracture, even after relatively minor falls [1]. Bone mineral density (BMD) is a parameter commonly used to measure bone strength and serves as a reliable indicator of osteoporosis. Higher body weight has been identified as a factor that is conducive to maintaining BMD [2–4]. While this association between obesity and BMD is widely recognized, there is still a disagreement regarding the impact of fat mass on bones [5–7]. Evidence has shown that body fat may have different effects on the BMD, depending on the region of deposition; this regional variation in the manner in which fat mass influences bone density may be attributed to the differences in the effects of subcutaneous fat and visceral adipose tissue (VAT) on bones. For example, in young, healthy men, trunk fat, rather than appendicular fat, is negatively correlated with cortical bone size [8]. Further, subcutaneous fat has been shown to have a positive correlation with the structure and strength of bones in females [9, 10]. A prospective cohort study revealed that excess visceral fat is associated with a high risk of nonspinal fractures in nonobese elderly women [11]. Moreover, Ng et al. found that this association was stronger among postmenopausal women [12].

Adult growth hormone deficiency (AGHD) is an endocrinal disorder caused by multiple factors, mainly pituitary tumors and/or their treatment [13]. When AGHD remains untreated, patients exhibit increased fat mass, especially visceral fat, and they have an increased risk of fractures [14]. Fleseriu et al. have shown that AGHD patients have a 2–5-fold increase in the risk of fracture [15]. The severity of GHD has a direct impact on the degree of bone loss and fracture risk. It compromises the patient's health-related quality of life and contributes to morbidity. Therefore, it is important to understand and evaluate the impact of AGHD on body composition. Although some studies have investigated the role of body composition in regulating bone density [16, 17], data on these mechanisms in AGHD patients are limited. Further investigations are necessary to understand the relationship between total fat mass, visceral fat, and BMD in AGHD patients.

Considering the lack of current knowledge in the manner in which fat mass (FM), especially visceral fat, contributes to BMD, we sought to examine the possible relationships between body composition parameters and BMD in AGHD patients.

## 2. Materials and Methods

### 2.1. Participants

We enrolled 57 patients (38 females and 19 males) diagnosed with AGHD in this study. The diagnosis of GHD in all the cases was established using the insulin tolerance test (ITT), which is the gold standard for the diagnosis of AGHD. All patients were investigated at the First Affiliated Hospital of Chongqing Medical University between February 2009 and December 2018. Patients who met the following criteria were excluded from the study: (1) levels of other pituitary hormone maintained within the normal reference range for at least the previous six months; (2) history of other systemic illness or medications known to affect bone metabolism, such as hyperthyroidism, rheumatoid arthritis, severe liver and kidney disease, and oral bisphosphonates; (3) history of musculoskeletal diseases, heart diseases, malignant tumor, or mental disorder; (4) state of pregnancy or lactation. All participants provided written informed consent, and the study protocol was approved by the ethics committee of the First Affiliated Hospital of Chongqing Medical University.

### 2.2. Anthropometry and Biochemical Measurements

All subjects underwent measurement of height, body weight, waist circumference, hip circumferences, and blood pressure test using the standard clinical procedures, while wearing light clothing and no shoes. Height and waist and hip circumferences were measured up to the nearest 0.1 cm, while body weight was determined with an accuracy of ±0.1 kg.

The patients were required to fast overnight, and their venous blood samples were collected to measure the values of the following biochemical parameters: fasting plasma glucose (FPG), total cholesterol (TC), triglyceride (TG), high-density lipoprotein cholesterol (HDL-C), and low-density lipoprotein (LDL-C). All the biochemical variables were determined by an automatic biochemical assay (Type 7600, Hitachi Ltd, and Japan). As per the guidelines for the prevention and treatment of dyslipidemia in Chinese adults, dyslipidemia was defined by the following: serum TG level of ≥1.70 mmol/L, serum TC level of ≥5.18 mmol/L, serum LDL-C level of ≥3.37 mmol/L, or serum HDL-C level of <1.04 mmol/L [18].

### 2.3. Assessment of Bone Mineral Density and Body Composition

Measurements of FM, VATM, and BMD of the lumbar spine (L1-L4) were obtained by a DXA system (Hologic Discovery QDR® Series, Bedford, MA, USA), which is a reliable tool for detecting osteoporosis. The device used was calibrated every morning, and its coefficient of variation (CV) for bone measurements was less than 1%. In addition, a whole body scan was performed with DXA to determine the FM and VATM, with a CV of <2.3%. BMD was expressed as T-score, and it was calculated by the National Health and Nutrition Examination Survey database, as provided by the manufacturer. According to the World Health Organization (WHO) guidelines, patients were classified into three categories by their T-score: T-score of ≥−1.0 standard deviations (SD), normal; T-score between −2.5 SD and −1.0 SD, with osteopenia, and T-score of ≤−2.5 SD, osteoporosis [19].

All the procedures employed in this study were consistent with the ethical standards of the First Affiliated Hospital of Chongqing Medical University and the 1964 Declaration of Helsinki and its later amendments or comparable ethical standards.

### 2.4. Index Calculation

BMI = weight (kg)/height (m)^2^

Waist-to-hip ratio (WHR) = waist circumference (cm)/hip circumference (cm).

### 2.5. Statistical Analyses

Data for continuous variables were represented as means ± standard deviation or medians (interquartile range), as appropriate. The variables were compared using the independent samples *t*-test and Mann–Whitney *U* test. The relationships between various demographic and laboratory parameters pertaining to BMD were assessed by linear correlation and linear regression. Statistical analyses were performed using the Statistical Package for the Social Sciences (SPSS) 17.0 (SPSS Inc., Chicago, IL). A *P* value (2-tailed) of <0.05 was considered significant.

## 3. Results

The clinical and biochemical characteristics of male and female patients with AGHD are presented in Table 1. The values of BMI, WC, WHR, and VATM were significantly higher in men than in women (all *P* < 0.05), whereas the values of BMD were higher in women than in men (*P* < 0.05). No such sex-based differences were noted in terms of age, SBP, DBP, FPG, TC, TG, HDL-C, LDL-C, and FM. Further, the incidence of hypertension (defined by a history of hypertension or systolic blood pressure (SBP) of ≥140 mmHg or diastolic blood pressure (DBP) ≥90 mmHg) was 39.5% in women and 36.8% in men. Diabetes, which was defined by a history of diabetes or fasting plasma glucose level of FPG ≥7.0 mmol/L, was confirmed in 15.8% and 21.1% of the female and male patients, respectively. As per the criteria defined for Chinese individuals, the incidence of dyslipidemia was 52.6% and 42.1% according to serum TG levels, 26.3% and 21.1% according to serum TC levels, 31.6% and 26.3% according to serum LDL-C levels, and 39.5% and 36.8% according to serum HDL-C levels, in women and men, respectively.

Of the 57 AGHD subjects, 5 (8.8%; male *n* = 0, female *n* = 5) were normal, while 21 (36.8%; male *n* = 5, female *n* = 16) had osteopenia and 31 (54.4%; male *n* = 14, female *n* = 17) had osteoporosis according to their *T*-score values. Accordingly, male and female patients were classified into those with osteoporosis and those without osteoporosis (normal subjects and patients with osteopenia) (Table 2). Intriguingly, only female AGHD patients with osteoporosis had significantly higher values of FM and VATM, as compared to those without osteoporosis (both *P* < 0.05). On the other hand, male AGHD patients with osteoporosis had higher LDL-C values than those without osteoporosis.

Simple correlations between the various variables and BMD values were then derived by the Pearson correlation analysis. As shown in Table 3, the parameters of age, BMI, FM, and VATM showed a negative correlation with BMD in female patients with AGHD (all *P* < 0.05). However, no significant correlation between BMD and other variables was noted in male patients with AGHD.

Therefore, BMD was regarded as a dependent variable.  Table 4 shows the results of multiple linear stepwise regression analysis of the relationship between the independent variables and BMD by gender. The analysis demonstrated that VATM was closely associated with the BMD in females, even after correcting for other covariates, including age, BMI, and FM.

## 4. Discussion

This study was mainly aimed at investigating the relationship between the parameters indicating body composition and BMD in AGHD patients. Our major findings are as follows: first, AGHD patients had a considerably high incidence of risk factors of cardiovascular diseases, such as diabetes, hypertension, and hypertriglyceridemia. Second, the prevalence of low BMD was very high among AGHD patients. Third, only in female AGHD patients, the values of FM and VATM were significantly higher in patients with osteoporosis than in those without osteoporosis. Fourth, multiple linear regression analysis revealed that VATM was independently correlated to the BMD in female with AGHD.

AGHD patients are known to have a high risk of cardiovascular diseases [20–22]. This was also confirmed in our study. These results highlight the importance of screening AGHD patients for cardio-metabolic risk factors.

Apart from promoting growth, GH is known to affect body composition via various mechanisms such as glycolipid metabolism as well as bone formation and remodeling [23]. GH promotes the longitudinal bone growth in children and plays an important role in bone mass maintenance in adults. In addition, GH stimulates the activity of chondrocytes and osteoblasts and influences the proliferation and differentiation of their precursors [24, 25]. Furthermore, GH regulates the secretion of parathyroid hormone, thereby affecting bone remodeling. The development and function of several skeletal cell lineages are also regulated by insulin-like growth factor 1 (IGF-1) [26, 27]. Our findings suggest that patients with AGHD have low BMD and a high incidence of osteoporosis. Therefore, both the prevention and treatment of osteoporosis are important in AGHD patients to reduce the resultant morbidity and mortality.

In this study, we found that FM, VATM, and BMD correlated significantly in female AGHD patients, but not in male patients. The association between FM and BMD has been confirmed in several studies [28–30]. Moreover, the marked sex differences in the impact of FM on BMD have also been reported previously. In addition, the association between fat tissue and BMD has been shown to exist only in females, not in male. These sex-based discrepancies can be attributed to the differences in the sex hormone levels of males and females [31, 32]. Further, the association between FM and BMD in females may be due to the changes in the biomechanical forces and the increased levels of endogenous estrogen in women with higher FM. These higher levels of endogenous estrogen are the result of the aromatization of androgen to weak estrogen in subcutaneous fat tissue [33, 34]. The differential effect of fat distribution on BMD has been shown in several previous studies [35–37]. Patients with AGHD often exhibit central obesity, which is mainly caused by an increase in visceral fat. Put together, these findings explain why FM is negatively correlated with BMD in women with AGHD. Our study also revealed an inverse association between VATM and BMD in female patients with AGHD.

Our study has a few limitations. The present study has a cross-sectional design, which does not allow for the analysis of the causal relationships among the various parameters assessed. Therefore, long-term investigations are necessary to elucidate the changes in the relationship between body composition and bone density in an individual. Another limitation of our study is that the relatively small number of patients enrolled in study could affect the external validity. More large-scale studies are therefore warranted to confirm our findings.

In conclusion, we found that VATM is closely correlated with the BMD in women with AGHD. These results suggest that the accumulation of visceral adipose tissue might disrupt the bone homeostasis in this specific subpopulation.

## Figures and Tables

**Table 1:** Clinical and biochemical characteristics by gender.

Characteristics	Male	Female	*P* value
Age (years)	55.63 ± 15.14	52.84 ± 13.96	0.498
BMI (kg/m^2^)	25.16 ± 4.06	22.81 ± 3.41	**0.026**
WC (cm)	92.55 ± 9.93	81.87 ± 8.53	**<0.001**
WHR	0.93 ± 0.05	0.88 ± 0.06	**0.004**
SBP (mmHg)	120.95 ± 22.96	124.27 ± 18.62	0.323
DBP (mmHg)	82.58 ± 13.60	74.81 ± 14.41	0.057
FPG (mmol/L)	6.01 ± 1.83	6.10 ± 2.12	0.961
TC (mmol/L)	4.48 ± 1.16	4.75 ± 1.73	0.552
TG (mmol/L)	1.59 (0.87–2.34)	1.64 (1.09–3.08)	0.630
HDL-C (mmol/L)	1.15 ± 0.44	1.39 ± 0.53	0.299
LDL-C (mmol/L)	2.75 ± 1.18	3.12 ± 1.33	0.334
FM (g)	22050.13 ± 7371.52	19133.73 ± 6553.14	0.155
VATM (g)	705.56 ± 270.37	480.91 ± 253.43	**0.005**
BMD (T-score)	−2.74 ± 0.91	−2.12 ± 0.94	**0.021**

Data are expressed as the mean ± standard deviation (SD) or median (interquartile range). The values of parameters showing significant differences between the two genders are in bold (*P* < 0.05). *N* = 57 patients (38 females and 19 males). T-score of BMD was determined for the lumbar spine, at L1-4.

**Table 2:** Comparison of characteristics between the N-OP and OP groups by gender.

Characteristics Male	N-OP	OP	*P* value
*N*	5	14	
Age (years)	56.00 ± 9.95	55.50 ± 16.93	0.952
BMI (kg/m2)	23.05 ± 2.43	25. 91 ± 4.32	0.182
WC (cm)	86.20 ± 4.21	94.82 ± 10.50	0.096
WHR	0.90 ± 0.03	0.94 ± 0. 06	0.156
SBP (mmHg)	133.00 ± 12.10	128.86 ± 26.08	0.740
DBP (mmHg)	93.20 ± 9.18	78.79 ± 13.09	0.138
FPG (mmol/L)	6.30 ± 2.46	5.92 ± 1.76	0.767
TC (mmol/L)	3.80 ± 0.54	4.67 ± 1.23	0.193
TG (mmol/L)	0.64 (0.49–2.28)	1.74 (1.07–2.51)	0.347
HDL-C (mmol/L)	1.30 ± 0.43	1.11 ± 0.44	0.443
LDL-C (mmol/L)	2.08 ± 0.34	2.95 ± 1.28	**0.036**
FM (g)	18318.14 ± 4069.99	23485.52 ± 7963.91	0.191
VATM (g)	563.80 ± 147.27	760.08 ± 291.10	0.175
BMD (*T*-score)	−1.42 ± 0.24	−3.21 ± 0.48	**<0.001**


Female			
*N*	21	17	
Age (years)	53.38 ± 12.71	52.19 ± 15.86	0.801
BMI (kg/m^2^)	22.29 ± 3.55	23.48 ± 3.20	0.299
WC (cm)	81.43 ± 8.08	93.47 ± 7.90	0.727
WHR	0.88 ± 0.05	0.88 ± 0. 06	0.869
SBP (mmHg)	122.19 ± 18.34	127.00 ± 19.21	0.444
DBP (mmHg)	75.14 ± 14.91	74.38 ± 14.19	0.875
FPG (mmol/L)	5.53 ± 2.31	6.60 ± 2.17	0.518
TC (mmol/L)	5.16 ± 1.68	4.26 ± 1.72	0.128
TG (mmol/L)	1.62 (1.29–2.84)	1.92 (0.94–3.80)	0.529
HDL-C (mmol/L)	1.35 ± 0.48	1.45 ± 0.32	0.747
LDL-C (mmol/L)	2.92 ± 0.76	3.37 ± 1.82	0.385
FM (g)	16881.19 ± 4155.95	22029.84 ± 7984.97	**0.006**
VATM (g)	368.76 ± 157.84	608.80 ± 284.91	**<0.001**
BMD (*T*-score)	−1.40 ± 0.55	−3.00 ± 0.41	**<0.001**

Data are expressed as the mean ± standard deviation (SD) or median (interquartile range). The values of parameters showing significant differences between the two groups are marked in red. (*P* < 0.05). OP: osteoporosis group; N-OP: nonosteoporosis group. *T*-score of BMD was determined for the lumbar spine, at L1-4.

**Table 3:** Correlation between different variables and BMD by univariate analysis.

Characteristics	Male		Female	
	Correlation coefficient	*P*	Correlation coefficient	*P*
Age (years)	−0.099	0.686	−0.330	**0.013**
BMI (kg/m^2^)	−0.209	0.390	−0.338	**0.041**
WC (cm)	−0.284	0.238	−0.228	0.174
WHR	−0.344	0.149	−0.210	0.211
SBP (mmHg)	0.274	0.257	−0.278	0.095
DBP (mmHg)	−0.166	−0.397	−0.138	0.416
FPG (mmol/L)	−0.069	0.823	−0.046	0.822
TC (mmol/L)	−0.335	0.174	−0.127	0.467
TG (mmol/L)	−0.253	0.311	−0.196	0.273
HDL-C (mmol/L)	0.165	0.512	0.040	0.823
LDL-C (mmol/L)	−0.275	0.269	−0.273	0.118
FM (g)	−0.186	0.460	−0.408	**<0.001**
VATM (g)	−0.433	0.228	−0.506	**<0.001**

Data are expressed as the mean ± standard deviation (SD) or median (interquartile range). The values of the parameters that showed significant correlation with BMD are marked in red. (*P* < 0.05). BMD was measured in the ROI of the lumbar spine, at L1−4.

**Table 4:** Independent variables related to BMD in female AGHD patients by multivariate analysis.

Variables	Unstandardized coefficients		Standardized coefficients	*t*	*P*
	B	SE			
VATM	−0.306	0.074	−0.418	−4.203	**<0.001**

BMD values were quantified in the specific ROI of the lumbar spine, at L1−4, by DXA.

## Data Availability

The data used to support the findings of this study are available from the corresponding author upon request.
